# Pan-Cancer Analysis of Glycolytic and Ketone Bodies Metabolic Genes: Implications for Response to Ketogenic Dietary Therapy

**DOI:** 10.3389/fonc.2021.689068

**Published:** 2021-10-07

**Authors:** Liyuan Qian, Yunzheng Li, Yajuan Cao, Gang Meng, Jin Peng, Huan Li, Ye Wang, Tiancheng Xu, Laizhu Zhang, Beicheng Sun, Binghua Li, Decai Yu

**Affiliations:** Department of Hepatobiliary Surgery, The Affiliated Drum Tower Hospital, Medical School of Nanjing University, Nanjing, China

**Keywords:** warburg effect, ketone body metabolism, ketogenic diets, biomarker, metabolic subtypes

## Abstract

**Background:**

The Warburg effect, also termed “aerobic glycolysis”, is one of the most remarkable and ubiquitous metabolic characteristics exhibited by cancer cells, representing a potential vulnerability that might be targeted for tumor therapy. Ketogenic diets (KDs), composed of high-fat, moderate-protein and low carbohydrates, are aimed at targeting the Warburg effect for cancer treatment, which have recently gained considerable attention. However, the efficiency of KDs was inconsistent, and the genotypic contribution is still largely unknown.

**Methods:**

The bulk RNA-seq data from The Cancer Genome Atlas (TCGA), single cell RNA sequencing (scRNA-seq), and microarray data from Gene Expression Omnibus (GEO) and Cancer Cell Line Encyclopedia (CCLE) were collected. A joint analysis of glycolysis and ketone bodies metabolism (KBM) pathway was performed across over 10,000 tumor samples and nearly 1,000 cancer cell lines. A series of bioinformatic approaches were combined to identify a metabolic subtype that may predict the response to ketogenic dietary therapy (KDT). Mouse xenografts were established to validate the predictive utility of our subtypes in response to KDT.

**Results:**

We first provided a system-level view of the expression pattern and prognosis of the signature genes from glycolysis and KBM pathway across 33 cancer types. Analysis by joint stratification of glycolysis and KBM revealed four metabolic subtypes, which correlated extensively but diversely with clinical outcomes across cancers. The glycolytic subtypes may be driven by TP53 mutations, whereas the KB-metabolic subtypes may be mediated by CTNNB1 (β-catenin) mutations. The glycolytic subtypes may have a better response to KDs compared to the other three subtypes. We preliminarily confirmed the idea by literature review and further performed a proof-of-concept experiment to validate the predictive value of the metabolic subtype in liver cancer xenografts.

**Conclusions:**

Our findings identified a metabolic subtype based on glycolysis and KBM that may serve as a promising biomarker to predict the clinical outcomes and therapeutic responses to KDT.

## Introduction

Metabolic reprogramming is a well-established hallmark of cancer (1). Malignant cells rewire their pathways of nutrient acquisition and metabolism to satisfy their bioenergetic, biosynthetic, and redox demands, and to further promote their growth, survival, proliferation, and long-term maintenance (2–4). In contrast to normal differentiated cells, which rely primarily on mitochondrial oxidative phosphorylation (OXPHOS) to generate the energy needed for cellular processes, most cancer cells instead rely on aerobic glycolysis and lactic acid fermentation to produce energy regardless of oxygen levels, a phenomenon termed Warburg effect (5). Warburg hypothesized that such metabolic preference was a consequence of dysfunctional mitochondria (6). Over the last decade, considerable progress in the field has advanced our understanding of the Warburg effect (7–9). Although it still remains unclear whether the Warburg effect plays a causal role in cancers or it is a consequence of genetic dysregulation during tumorigenesis, therapies targeting the Warburg effect are emerging and promising (10).

Ketogenic dietary therapy (KDT) has gained substantial attention in recent years as an alternative treatment strategy to target altered glucose metabolism in cancer cells (11). Ketogenic diets (KDs) are composed of high fat, moderate protein and low carbohydrates, which favors mitochondrial respiration rather than glycolysis for energy metabolism, such that cancer cells are selectively starved of energy (12). The mechanism by which KDs demonstrate anticancer effects has not been fully elucidated (13). Evidence suggests that ketone body is the bioactive metabolite of KDs, which is necessary and sufficient to account for the anti-cancer effect of KDs (14). Although numerous preclinical studies indicated a therapeutic potential for KDs in cancer treatment, it is now becoming clear that not all tumors might respond positively (11, 15). It has been found that certain tumors maintain oxidative metabolism during tumor progression (16–18), and some cancer cells redirect to use ketone bodies under a certain condition (19, 20). Therefore, it is crucial to identify patients who may or may not respond optimally to KDT, as the efficacy of the KDT strongly depends on the tumor entity and its genotype (21).

Since KDs target the metabolic shift from OXPHOS to glycolysis in tumor cells, it is rational to hypothesize that the tumors with high glycolytic enzyme and low ketolytic enzyme expression may benefit from KDT, whereas tumor cells with highly expressed ketolytic enzymes may actively consume ketone bodies as an energy source, leading to the KDT resistance. Indeed, several studies have revealed that the therapeutic efficacy of KDs is influenced by the expression of metabolic enzymes, such as BDH1, OXCT1, and ACAT1 (22–25). However, these studies suffered from limited cancer types and genes. The growing application and integration of high-throughput measurements provide an opportunity to investigate a wider scope of dysregulated metabolism across different cancer types.

In this study, we comprehensively surveyed the expression profile and prognostic value of glycolysis and ketone bodies metabolism (KBM) genes in 33 The Cancer Genome Atlas (TCGA) cancer types. We characterized the metabolic subtypes in more than 10,000 tumor samples and nearly 1,000 Cancer Cell Line Encyclopedia (CCLE) cancer cell lines based on the expression pattern of glycolysis and KBM genes. We found that metabolic expression subtypes showed extensive heterogeneity across cancer types, and harbored different somatic mutations. Finally, we demonstrated that the glycolytic subtypes, which were associated with TP53 mutations, may be more likely to benefit from KDT, and further validated the predictive value of the metabolic subtypes in KDT using HCC xenograft models.

## Methods

### Data Sources

All RNA-seq gene expression data, somatic mutation data and clinical data of TCGA were downloaded from Xena (https://xenabrowser.net/datapages/). Only the samples with clinical information available were kept for the following analysis. As a result, a total of 11,031 samples including 719 non-tumor tissues and 10,312 tumor tissues representing 33 cancer types were included. Microarray dataset GSE36133 (from CCLE project) (26) was collected to analyze the metabolic pattern of cancer cell lines. Two single-cell datasets [GSE109774 (27) and GSE103867 (28)], which contain nearly 100,000 cells from 20 mouse organs and two HCC cell lines (HuH-1 and HuH-7), separately, were used to validate the expression patterns of glycolysis and KBM at the single-cell level in normal tissue and tumor cell lines.

### Curation of Glycolytic and Ketone Bodies Metabolic Gene Signatures

Gene sets involved in glycolysis and KBM were initially retrieved from the Molecular Signatures Database v 7.1 (MSigDB) (29). Three pathways regarding glycolysis from KEGG, REACTOME, and HALLMARK were taken into consideration. The intersected 11 genes among these three gene sets were used as marker genes for glycolysis. Then, we searched the MSigDB for ketone bodies related gene sets and reviewed the genes manually. The 10 genes from the Reactome_ketone_body_metabolism gene set were most reasonable and considered as ketone bodies metabolic (KBM) gene signatures (**Figure S1**).

### Single-Sample Gene Set Enrichment Analysis (ssGSEA)

Single-Sample Gene Set Enrichment Analysis (ssGSEA) is an extension of GSEA, which calculates separate enrichment scores for each pairing of a sample and gene set (30). The ssGSEA score was calculated using the Bioconductor GSVA package with the aforementioned glycolysis or KBM gene signatures, which was further used to evaluate the metabolic activities of glycolysis or KBM pathway in tumor tissues or cell lines.

### Classification of Metabolic Expression Subtypes Based on Glycolysis and KBM

The ssGSEA score was further transformed by Z-score for glycolysis and KBM among samples in each dataset. Patients or cancer cell lines were assigned to four metabolic subtypes based on the ssGSEA Z-score: the glycolytic subtype (glycolysis ≥0, KBM < 0), the KB-metabolic subtype (glycolysis<0, KBM ≥0), the mixed subtype (glycolysis<0, KBM ≥0), and inactive subtypes (glycolysis<0, KBM <0).

### Differential Analysis

The Bioconductor package edgeR was used to determine differential expression between non-tumor and tumor tissues at the RNA level and calculate the foldchange and FDR value across cancer types.

### Survival Analysis

The hazard ratio (HR) was estimated using a Cox regression model using the survival R package. Survival analysis was carried out using Kaplan-Meier methods and the log-rank test was used to determine the statistical significance of differences. The survival curve was generated by the R survminer package.

### Cell Line and Culturing

The human HCC cell line HuH-7 and SK-HEP-1 were cultured in DMEM supplemented with 10% fetal bovine serum, 100 U/mL penicillin and 100 μg/ml streptomycin (all from Thermofisher, NY, USA). These cell lines were authenticated by short tandem repeat (STR) analysis and tested for mycoplasma contamination. Cells were maintained at 37°C in a humidified atmosphere with 5% CO2 and maintained in culture for a maximum of 20 passages (two months).

### Animal Studies

Male BALB/c nude mice (6 weeks old) were obtained from the Model Animal Research Center of Nanjing University (Nanjing, China). All experimental procedures using animals were in accordance with the guidelines provided by the Animal Ethics Committee of the Affiliated Drum Tower Hospital of Medical School of Nanjing University. SK-HEP-1 or HuH-7 cells were subcutaneously injected into the flank of nude mice. The tumors were allowed to grow until they were palpable before initiating each treatment. The mice were randomly divided into two groups. The control group was fed the normal diet (ND), whereas the treatment group was fed a ketogenic diet (KD). The ketogenic diet was purchased from Dyets (http://www.dyets-cn.com/, #HF89.5), which consisted of 0.1% carbohydrates, 89.5% fat, and 10.4% protein. Tumor growth was determined by measuring the short and long diameter of the tumor with a caliper every three days. 5-6 weeks after injection, the mice were sacrificed. Tumor volume was calculated according to the formula volume = width × width × length/2.

### Blood Biochemical Level Measurements

Blood samples were collected before sacrificing the mice. Serum levels of alanine aminotransferase (ALT), aspartate aminotransferase (AST), high-density lipoprotein (HDL), low-density lipoprotein (LDL) and total cholesterol (TC) and triglyceride (TG) were measured using an automated chemical analyzer in the department of Laboratory Medicine, The Affiliated Drum Tower Hospital.

### Western Blot

Western blot was performed as previously described (31). Briefly, total proteins of tumor tissues were isolated with RIPA buffer (#P0013C, Beyotime) and the concentrations of which were measured using a BCA detecting kit (#P0012, Beyotime). The lysates were fractionated by SDS PAGE and transferred to PVDF membranes. Primary and secondary antibodies were used to detect the targets on the membranes. The primary antibodies used were: anti-OXCT1 (#12175-1-AP, Proteintech), anti-HMGCS2 (#ab137043, Abcam), anti-ENO2 (#10149-1-AP, Proteintech), anti-PKM2 (#15822-1-AP, Proteintech), anti-HK2 (#22029-1-AP, Proteintech), anti-β-actin (#4970s, CST). The quantitative analysis was performed using Photoshop.

### Statistical Analyses

All statistical analyses were performed using R language. Student’s t-test or Wilcoxon rank-sum test were used to compare the median values of two sets of continuous variables. The count data were analyzed with Pearson Chi-Square. Correlation between two continuous variables was measured by either Pearson’s r correlation or Spearman’s rank-order correlation. The repeated-measures ANOVA was used to compare the difference of the tumor volume in different groups. The two-sided p-value less than 0.05 was defined as statistically significant for all statistical analyses. The data were plotted as mean ± standard deviation (SD).

## Results

### A Systematic Landscape of the Expression Pattern and Prognosis of Glycolysis and KBM Signature Genes

**Figure 1:**
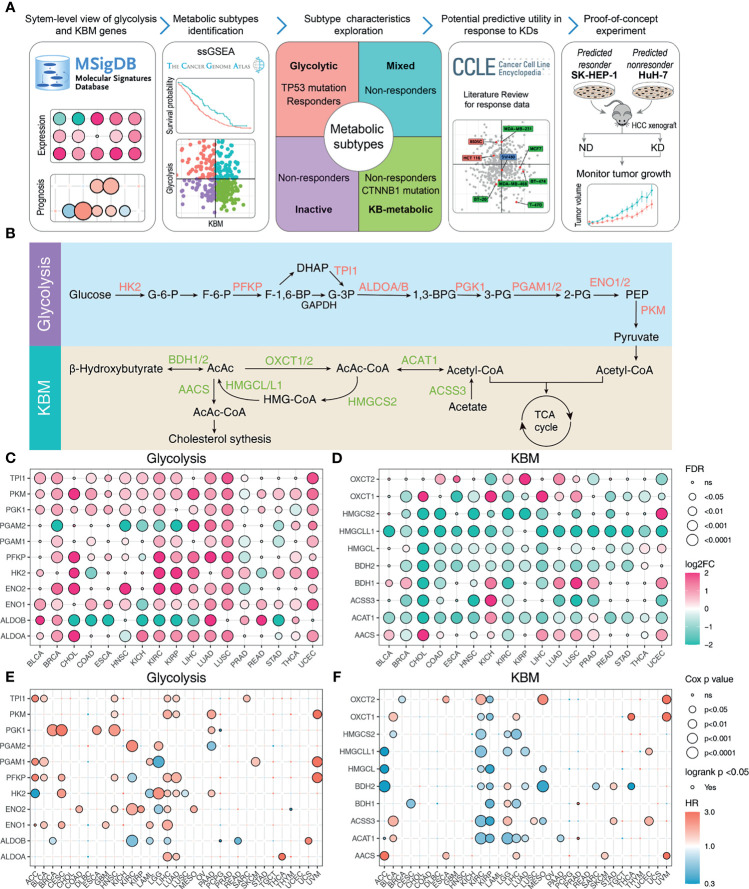
The expression pattern and prognosis of glycolysis and KBM genes in cancer patient samples from TCGA datasets. **(A)** Overview of the study workflow. **(B)** Schematic model illustrating the glycolysis and KBM pathway. The selected signature genes are marked in the pathway, with glycolytic enzymes in red and ketone bodies metabolic genes in green. The bubble plot shows the expression pattern of glycolysis **(C)** and KBM **(D)** genes in non-tumor and tumor tissues across cancer types. Only the cancer types with at least 5 non-tumor tissues were included in this analysis. Red represents upregulation and green represents downregulation. The point size represents fold-change and the shade of color represents p-value. The FDR and fold change were determined by edgeR. The bubble plot shows the result of survival analysis of glycolysis **(E)** and KBM **(F)** genes for each cancer type. Both Cox proportional analysis and log-rank test were used to determine the statistical significance, which was represented by the size and border of circle, respectively. The color of circle represents hazard ratio (HR), in which red indicates high risk for survival and green indicates low risk. The p-value was determined by log-rank test and wald test. ns, not significanct.

The flow chart of our study design is displayed in **Figure 1A**. We firstly explored the Molecular Signatures Database (MSigDB) to identify glycolysis and KBM signature genes. The intersected 11 genes among the glycolysis pathway from the KEGG, Reactome, and Hallmark annotations were considered as the glycolysis signature (**Figure S1A**). We also compared the curated KBM gene sets from MSigDB. The gene set named Reactome Ketone Body Metabolism represented the most reasonable summary of the KBM pathway, thus all the 10 genes from this gene set were selected as KBM signature (**Figure S1B**). These 21 genes were projected onto the metabolic pathways to obtain a systematic view and used for the following analyses (**Figure 1B**).

To gain a more comprehensive view of the expression pattern of glycolysis and KBM signature genes, 17 cancer types with matched non-tumor samples available were evaluated. Overall, most glycolysis and KBM signature genes were differentially expressed across cancer types, with glycolysis signature genes upregulated (**Figure 1C**), whereas KBM signature genes downregulated in most cancer types (**Figure 1D**). To further clarify the involvement of abnormal glycolysis and KBM in tumor progression, the correlations between signature genes and outcomes were assessed using both Cox analysis and log-rank analysis. Their associations with patient survival varied significantly by different cancer types, in which most glycolysis signature genes were associated with worse survival (**Figure 1E**). In contrast, most KBM signature genes are favorable in multiple cancer types (**Figure 1F**). These analyses provide a broad view of glycolysis and KBM signature genes in human cancer.

### The Metabolic Activities of Glycolysis and KBM in Normal and Tumor Tissues and Their Associations With Clinical Outcomes

**Figure 2:**
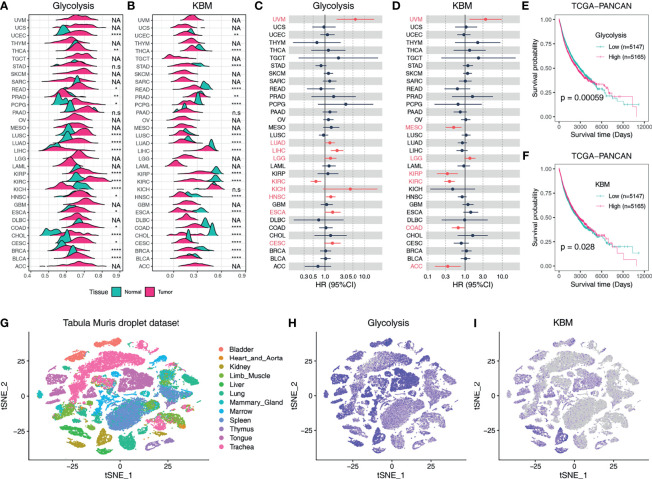
The expression profiles and clinical relevance of glycolysis and KBM activity based on ssGSEA score. Ridgeline plot shows the metabolic activity of glycolysis **(A)** and KBM **(B)** pathway in non-tumor tissues and tumor tissues across TCGA cancer types based on ssGSEA score. The p-value was determined by Student’s t-test. Forest plots show the hazard ratio (HR) of overall survival of the glycolysis **(C)** and KBM **(D)** ssGSEA score across cancer types. The cancer types marked in red indicated Cox proportional p-value <0.1. Kaplan-Meier plots for glycolysis **(E)** and KBM **(F)** ssGSEA score in TCGA Pan-Cancer (PANCAN) dataset. ssGSEA is stratified by the median value in each cancer type individually, then combined to generate the survival curve. The p value was determined by log-rank test. **(G)** t-SNE plot of all cells in Tabula Muris droplet dataset, colored by organ. t-SNE plots showing expression and distribution of glycolysis **(H)** and KBM **(I)** ssGSEA score across organs. *p < 0.05; **p < 0.01; ****p < 0.0001. n.s., not significant; NA, not available.

The metabolic activities of pathways can be reflected by the expression patterns of metabolic genes as previously described (32). We next aimed to characterize the metabolic activities of glycolysis and KBM based on the expression pattern of their signature genes using the ssGSEA method. Glycolysis was upregulated in most tumors compared to matched non-tumor tissues as calculated by ssGSEA score (**Figure 2A**). On the contrary, KBM was downregulated in many cancer types (**Figure 2B**). A weak negative correlation was observed between glycolysis and KBM (**Figure S2A**). Besides, KBM was highly expressed in normal liver and kidney tissue, which is consistent with the finding that KBM primarily takes place in the liver and kidney (33). Associations with patient outcomes were examined for every cancer type individually. High glycolysis activity indicated a higher death risk in eight TCGA cancer types (HR>1, **Figure 2C**), whereas high KBM metabolic activity had a favorable effect in five cancer types (HR <1, **Figure 2D**). Notably, both glycolysis and KBM were negatively correlated with patient prognosis in UVM and LGG, indicating that these cancers may have unique metabolic features.

We further combined all cases of the 33 different cancer types into one dataset (PANCAN) and the Kaplan-Meier curves were utilized to display patient survival for all TCGA cases (34). Overall, higher glycolysis activity contributes to poorer outcomes (**Figure 2E**), whereas higher KBM patients had prolonged overall survival (**Figure 2F**). Patients were classified into four categories based on glycolysis and KBM levels. Having a high level of glycolysis and a low level of KBM resulted in reduced overall survival compared to other groups (**Figure S2B**). We then analyzed the metabolic activities of glycolysis and KBM at the single-cell level across normal organs using the Tabula Muris dataset (**Figure 2G**). Glycolysis genes were broadly expressed in most mouse organs (**Figure 2H**), while KBM was constricted to the liver and kidney, which is consistent with the bulk RNA-seq from TCGA dataset (**Figure 2I**).

### Metabolic Expression Subtypes Show Extensive Heterogeneity Across Cancer Types

**Figure 3:**
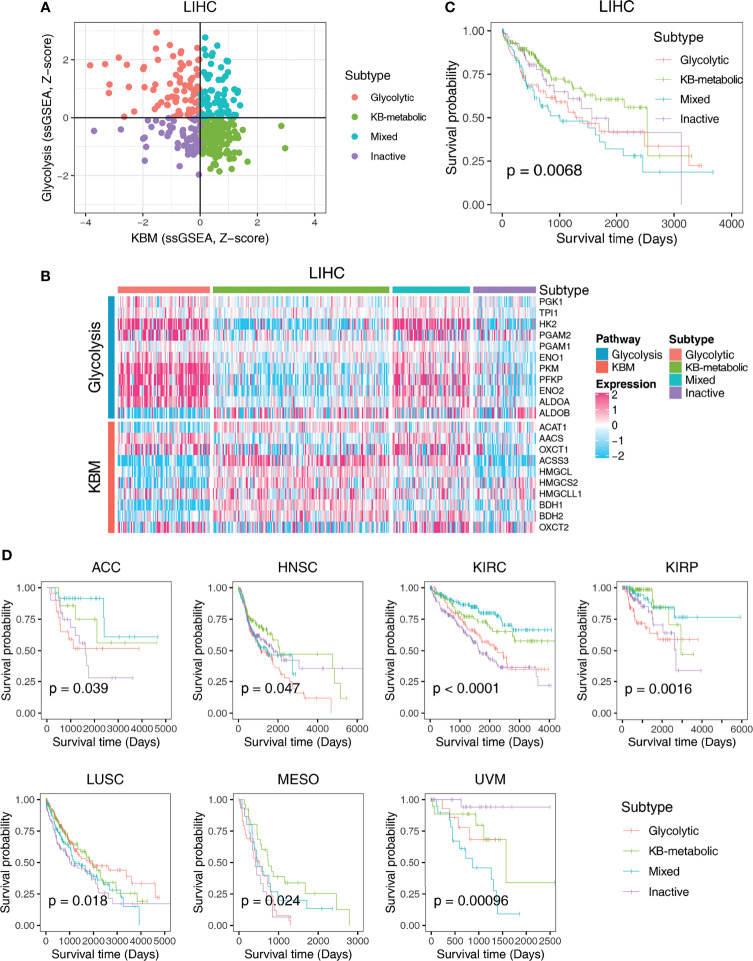
Classification of metabolic expression subtypes and their associations with patient survival time. **(A)** Scatter plot of the ssGSEA score of KBM (x-axis) and glycolysis (y-axis) genes in each LIHC sample. Metabolic subgroups were assigned based on the relative levels of glycolysis and KBM genes. **(B)** Heatmap showing the expression pattern of glycolysis and KBM genes in each subtype. **(C)** Kaplan-Meier survival analyses of patients with different subtypes of LIHC. **(D)** The survival curves of different metabolic subtypes in different cancer types. The p-value was determined by log-rank test.

Since the correlations between glycolysis or KBM and patient overall survival differ widely across cancer types (**Figures 2C, D**), we next aimed to characterize metabolic heterogeneity within each cancer type based on the metabolic activity of glycolysis and KBM. Taken LIHC dataset as an example, hepatocellular carcinoma (HCC) patients were divided into four subtypes based on the co-expression of glycolysis and KBM score: the glycolytic subtype (high glycolysis score and low KBM score), the KB-metabolic subtype (high KBM score and low glycolysis score), the mixed subtype (high of both glycolysis and KBM score), and the inactive subtype (low of both glycolysis and KBM score) (**Figures 3A, B**). The KB-metabolic subtype had the best prognosis, and the mixed subtype had the worst clinical outcome in HCC (**Figure 3C**). We further explored the clinical relevance of metabolic subtypes across cancer types. Glycolytic subtype had worse overall survival than patients from other subtypes in ACC and KIRP. KB-metabolic subtype patients tend to have a better prognosis in HNSC and MESO. The mixed subtypes had the worst outcome in UVM. Conversely, they exhibited the longest survival time in KIRC (**Figures 3D** and **S3**). Overall, these results highlight the diverse effect of the involvement of metabolism subtypes in different cancer types.

### Metabolic Expression Subtypes Are Associated With Different Somatic Drivers

**Figure 4:**
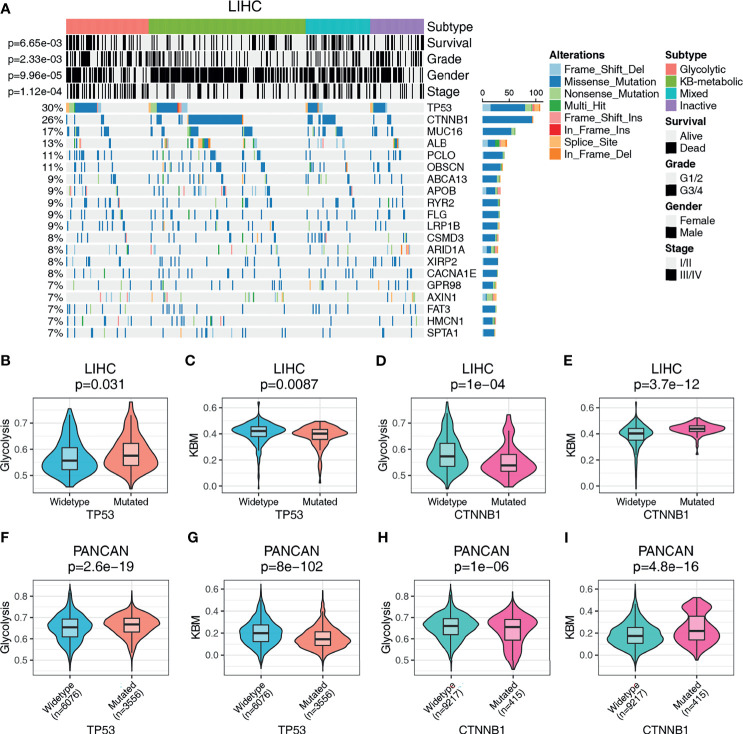
The genomic landscape of metabolic subgroups and mutational characteristics. **(A)** Oncoplot summarizing the distribution of SNVs and INDELs mutational frequency in LIHC across the metabolic subtypes for the top 20 most mutated genes. Top panel details gender, tumor grade, stage, and prognosis for HCC patients. The metabolic activity of glycolysis or KBM in TP53 or CTNNB1 wildtype or mutation patients in LIHC **(B–E)** or PANCAN dataset **(F–I)**. Student’s t-test or Wilcoxon rank-sum test were used to compare the median values of two sets of continuous variables. The count data were analyzed with Pearson Chi-Square.

The metabolic phenotype of tumor cells is largely controlled intrinsically by tumorigenic mutations (35). To identify genetic alterations that potentially drive the metabolic subtypes, we performed a correlation analysis and plotted the oncoplot of the top 20 mutated genes for each cancer type categorized by metabolic subtypes. We observed that glycolytic subtype harbors higher mutational ratio of TP53 [35/83 (42.2%) *vs.* 39/158 (24.7%), p=0.008], but a significant lower mutational ratio of CTNNB1 [5/83 (6.0%) *vs.* 65/158 (41.1%), p= 2.75e-08] than KB-metabolic subtype in HCC (**Figure 4A**). No other mutations showed differential distribution. Also, negative associations were detected between glycolytic subtypes and tumor grade (p= 2.33e-03), stage (p= 1.12e-04) and overall survival (p= 6.65e-03). Interestingly, we found glycolytic subtypes tended to be female in HCC patients (p= 9.96e-05). We further compared the metabolic activity of glycolysis and KBM (measured by ssGSEA score) in wide-type and mutated samples. HCC patients with mutated TP53 had much higher glycolysis (p=0.031, **Figure 4B**) and lower KBM metabolic activity (p=0.0087, **Figure 4C**) than wide-type patients. In contrast, patients with mutated CTNNB1 had lower glycolysis score (p=1e-04, **Figure 4D**) but higher KBM score (p=3.7e-12, **Figure 4E**).

Similarly, the phenomenon that glycolytic subtypes had much higher mutation frequency of TP53 was observed in ACC, HNSC and LUSC (**Figure S4**). Notably, no patients in the KB-metabolic subtype harbor TP53 mutations (0/14 *vs.* 7/21), with more patients bearing CTNNB1 mutations (3/14 *vs.* 2/21) than glycolytic subtype in ACC. Furthermore, we compared the glycolysis and KBM score in PANCAN dataset (n= 9632). TP53 mutations are positively associated with glycolysis score and negatively correlated to KBM score (**Figures 4F, G**). An opposite trend is presented in patients with CTNNB1 mutation, where CTNNB1 mutations were positively correlated with KBM but inversely correlated with glycolysis score (**Figures 4H, I**). Together, these results suggest that there are common features in genetic alterations related to the metabolic subtypes across cancer types and that TP53 mutations were associated with glycolytic subtype, while CTNNB1 mutations were correlated to KB-metabolic subtype.

### Metabolic Expression Subtypes Are Informative About the Response to Ketogenic Diet Therapy

**Figure 5:**
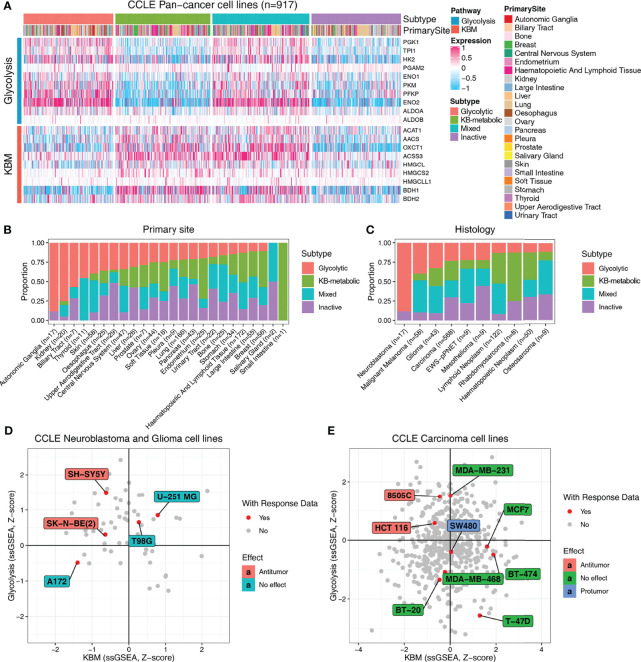
Metabolic subtypes based on glycolysis and KBM genes are informative about the response to KDT. **(A)** Heatmap presenting the expression pattern of glycolysis and KBM genes in each subtype across CCLE cancer cell lines. The proportion of four metabolic subtypes based on primary site **(B)** and histology **(C)** in the CCLE cell lines. The metabolic subtypes of neuroblastoma and glioma **(D)** and carcinoma cell lines **(E)** and their relationship with the reported response data of ketogenic therapy.

Next, we predicted the metabolic subtypes of individual cancer cell lines using CCLE data. In accordance with the results obtained from TCGA primary tumors, CCLE cell lines were categorized into four subtypes based on glycolysis and KBM activity, in which glycolytic subtypes highly expressed glycolysis signature genes and KB-metabolic subtypes had abundant KBM gene expression (**Figure 5A** and **Table S1**). The proportions of subtypes varied substantially across primary sites and histology. Cancer cell lines from the autonomic ganglia, kidney and biliary tract had the highest proportion of glycolytic subtype (**Figure 5B**). The neuroblastoma accounts for the highest proportion of glycolytic subtype compared to other histology (**Figure 5C**).

Based on the expression characteristics of glycolytic subtypes (high glycolysis and low KBM) and KB-metabolic subtypes (high KBM and low glycolysis), it seems reasonable to hypothesize that patients with high level of glycolytic enzymes and low expression of ketone bodies metabolic enzymes (glycolytic subtypes) may respond better to KDT. To test that hypothesis, we firstly systematically reviewed and curated the relevant literature with response information of KDT available from animal studies (**Table S1**). Neuroblastoma and glioma are the most extensively investigated cancer types of KDT. Therefore, we firstly analyzed all the neuroblastoma and glioma cell lines. Five human neuroblastoma and glioma cell lines had response data available, two of which are experimentally verified to be responders of KDs. Of note, both cell lines were identified as glycolytic subtypes in our study (**Figure 5D**). Similarly, KDs are reported to have an anti-tumor effect in 8505C, a thyroid carcinoma cell line, as well as HCT 116, a large intestine cell line, both of which are identified as glycolytic subtypes in our study. Likewise, KDs are proved to have either no effect or even protumor effect in carcinoma cell lines from other subtypes (**Figure 5E**). Collectively, these data indicated that the metabolic subtypes may serve as potentially useful biomarkers to select patients who may potentially yield clinical benefits to KDT.

### Validating the Predictive Utility of Metabolic Subtypes in Response to KDT Using the HCC Xenograft Model

**Figure 6:**
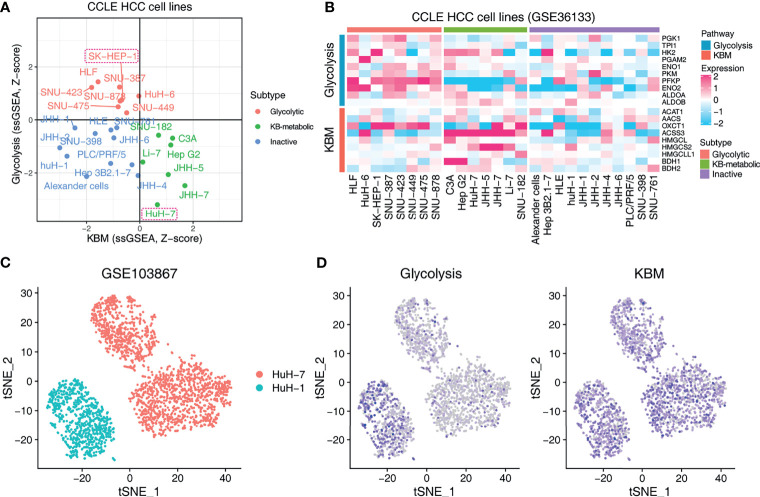
Exploring the metabolic subtypes in HCC cell lines. **(A)** Scatter plot showing the subtype of CCLE HCC cell lines. Red indicates glycolytic subtype, and green indicates KB-metabolic subtype. The cell lines in the dashed boxes were chosen for the following experiment. **(B)** Heatmap showing the expression pattern of glycolysis and KBM genes of CCLE HCC cell lines with different subtypes. **(C)** t-SNE plot of HuH-1 and HuH-7 from GSE103867 dataset. **(D)** The pathway activity of glycolysis and KBM represented by ssGSEA score in t-SNE space from cells in **(C)**.

**Figure 7:**
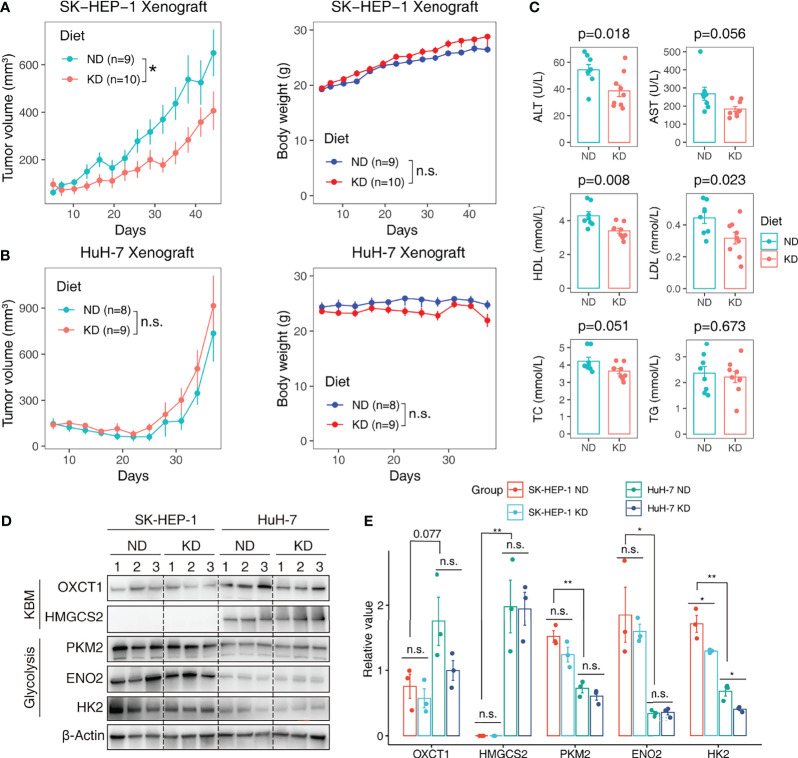
Validating the predictive value of metabolic subtypes in KDT in HCC xenograft model. HCC cell line SK-HEP-1 (glycolytic subtype) and HuH-7 (KB-metabolic subtype) were subcutaneously inoculated into the flank of nude mice. The tumor-bearing mice were random allocated to the normal diet (ND) or ketogenic diet (KD) group. Tumor volumes were monitored every three days. Tumor growth curve and body weight growth curve of SK-HEP-1 **(A)** and HuH-7 **(B)** were shown. **(C)** The serum ALT, HDL and TC level in ND and KD group mice. **(D)** Western blot analysis of glycolysis and KBM enzymes in SK-HEP-1 and HuH-7 tumors. Limited by the well number of SDS PAGE, we randomly test three samples from each group. **(E)** Quantification of protein levels normalized onto β-actin in each condition. Data are presented as mean ± SD. Student t-test was used for statistical analysis. *p < 0.05; **p < 0.01; n.s., not significant.

We further performed a proof-of-concept experiment to validate the predictive utility of the metabolic subtypes in KDT using xenograft models. Only three subtypes are predicted to be present in HCC cell lines (**Figure 6A**). We selected two representative lines, SK-HEP-1 (glycolytic subtype) and HuH-7 (KB-metabolic subtype) for *in vivo* examination of KD sensitivity (**Figure 6B**). The expression pattern of glycolysis or KBM genes in HuH-7 cells was further validated by a scRNA-seq dataset (**Figure 6C**). HuH-7 cells highly expressed KBM genes with low glycolysis gene expression (**Figure 6D**). SK-HEP-1 and HuH-7 cells were injected into the flank of nude mice. Mice were fed with normal diet (ND) or ketogenic diet and humanely sacrificed 5 weeks after treatment. As anticipated, SK-HEP-1 tumor volumes were markedly reduced in the KD group compared to the ND group (**Figure 7A**, left panel). However, the HuH-7 tumors in the KD group even had a trend to grow faster than that in the ND group (**Figure 7B**, left panel). No apparent signs of toxicity or side effect were observed as evidenced by no significant difference in the body weight (**Figures 7A, B**, right panel), ALT, AST, HDL, LDL, TC and TG between KD and ND group (**Figure 7C**), indicating that KD was well tolerated. Finally, we detected the enzymes participating in glycolysis and KBM in the tumor tissues. HMGCS2 and OXCT1, the rate-limiting enzyme of ketogenesis or ketolysis respectively (36), were expressed higher in HuH-7 tumors compared to SK-HEP-1 ones (**Figures 7D, E**). However, HK2, ENO2 and PKM2, the vital key enzymes in the metabolic process of glycolysis (37, 38), were highly expressed in SK-HEP-1 tumors (**Figures 7D, E**), which was consistent with the results obtained from big data (**Figures 6A, B**). Notably, except HK2, which was slightly decreased, there seems to be no change in the levels of other glycolysis and KBM enzymes we test after KDT (**Figures 7D, E**).

## Discussion

The Warburg effect is an extensively studied phenomenon characterized by increased aerobic glycolysis and excessive lactate formation (5). Numerous studies have shown that enhanced glycolysis predicts poor prognosis, promotes tumor progression, immune escape and drug resistance in different categories of cancers (39, 40). The classic Warburg effect describes the shift from OXPHOS to glycolysis, it should be noted that cancer cells can switch their metabolism phenotypes between glycolysis and OXPHOS during tumorigenesis and metastasis (41). Ketone bodies generated by fatty acid oxidation can serve as an alternative fuel for OXPHOS (42). Thus, it’s worthwhile to comprehensively characterize the glycolysis and KBM pathway. The connections between KBM and cancer are rapidly emerging (36, 43). However, a joint analysis of glycolysis and KBM is lacking. In this study, we performed the first systematical combined-analysis of glycolysis and KBM genes across cancers. Based on the expression profiles of these genes, we identified the glycolytic and KB-metabolic subtypes, which highly expressed the glycolysis or KBM signature genes respectively, and also characterized a hybrid metabolic subtype and an inactive subtype. The metabolic expression subtypes showed extensive heterogeneity in prognosis across cancer types. Surprisingly, the inactive subtypes, with low activity of both glycolysis and KBM, which were supposed to be low malignancy, tended to show a worse overall survival in KIRC, LUSC and MESO. A possible explanation for this might be that the inactive subtypes are not fully metabolically inactive, for other metabolic pathways might compensate for the low activity of glycolysis and KBM in these cancer types. Although the previous research has reported that expression patterns of metabolic genes could reflect metabolic activities in cancer patients (32), further study is still warranted to clarify the relationship between the level of metabolic gene expression and glycolysis or KBM pathway activities.

Theoretically ketogenic diet, which reduces glucose availability to tumor cells, while providing ketone bodies as an alternative bioenergetic fuel to normal cells, could result in selective starvation of tumor cells, for tumor cells are unable to adapt to ketone metabolism as a result of their acquired metabolic inflexibility and genomic instability (14). However, the efficiency of KD was inconsistent, with both the anti-tumor effects and pro-tumor effects were well-reported in preclinical and clinical studies (15). It is necessary to explore the sensitive indicators that predict or affect KD effectiveness. In this study, we found that the metabolic expression subtypes based on glycolysis and KBM activity are informative about the KDT. KDs reduced tumor growth and prolonged survival in the SH-SY5Y and SK-N-BE(2) xenografts (23, 44, 45), both of which were identified as the glycolytic subtype in our study. Three human glioma cell lines, which were either identified as mixed or inactive subtypes in our study, were proven to be non-responders to KDs (46). Similarly, our metabolic subtypes predicted the positive response to KDs in thyroid cancer (47) and large intestine cancer cell lines (48, 49). It has been reported that ketone bodies can behave as onco-metabolites and that ketone bodies utilization drives tumor growth and metastasis in breast cancers (19, 50). Interestingly, none of the cell lines were identified as glycolytic subtypes in our study. Thus, the metabolic subtypes are informative about the response to KDT, although these findings may be somewhat limited by the small number of cases. We further performed a proof-of-concept experiment to validate the predictive value of the metabolic subtype using liver cancer, a cancer type with conflicting responses to KDT was reported (51, 52). Consistently, KDs reduced the growth of the cell line belongs to the glycolytic subtype, but tended to promote the growth of the KB-metabolic subtype cell line. Therefore, the metabolic expression subtypes defined here have potential clinical implications in guiding personalized KDT, and further efforts will be required to validate the findings in other cancer types. Metabolic flexibility, which means the intrinsic ability of cells to change from one metabolic fuel source to another (53), is one of the most important characteristics of cancers (54). Thus, the adaptability of different cancer cells to metabolic changes cannot be ignored, which may affect the efficacy of metabolic therapy. It is a limitation to just consider the initial state of the cancer metabolism instead of the metabolic changes. Also, other metabolic signatures besides glycolysis and KBM may be of importance because of the metabolic adaptability.

Metabolic reprogramming may result from diverse somatic driver alterations. We explored the somatic mutations underlying the different subtypes. TP53 mutations were associated with glycolytic subtype, while CTNNB1 mutations were correlated to the KB-metabolic subtype. This observation may support the hypothesis that TP53-driven tumors are candidates for KDT, while β-catenin-driven tumors are in the contrast. Previous research has established that TP53 mutations increase glycolysis in multiple cancer types (55–59). Several attempts have also been made to illustrate the role of CTNNB1 and KBM. It has been reported that HMGCS2, a rate-limiting ketogenic enzyme in the synthesis of ketone bodies, was is a novel target of Wnt/β-catenin (60). Moreover, recent studies have shown that β-catenin-activated HCC were not glycolytic but intensively oxidized fatty acids. PPARα, the key transcription factor taking part in fatty acid oxidation and ketogenesis, is also a β-catenin target (33, 61). Therefore, β-catenin may control the ketogenesis process. In our study, we found that both ketogenesis and ketolysis enzymes were highly expressed in the KB-metabolic subtype. However, the relationship between β-catenin and ketolysis has not yet been deciphered. Continued efforts are needed to assess the broader role of β-catenin in KBM.

## Conclusions

In conclusion, we performed a joint analysis of glycolysis and KBM genes in this study. We identified four subtypes based on the activity of glycolysis and KBM pathway. The subtypes were correlated extensively but diversely with clinical outcomes and somatic mutations. Both literature review and a proof-of-concept experiment provide preliminary but exciting evidence supporting that our subtypes were informative about the response to KDT, and further studies both in the laboratory and clinically will still be warranted.

## Data Availability Statement

The datasets presented in this study can be found in online repositories. The names of the repository/repositories and accession number(s) can be found in the article/**Supplementary Material**.

## Ethics Statement

The animal study was reviewed and approved by the Animal Research Ethics Committee of the Affiliated Drum Tower Hospital of Nanjing University Medical School.

## Author Contributions

BL and DY designed the project. BL and YL performed the bioinformatic analysis. BL, LQ, and DY wrote the manuscript. LQ, GM, and TX conducted the experiments. YC, JP, HL, YW, and LZ assisted in performing experiments and manuscript revision. BS and DY supervised the project. All authors contributed to the article and approved the submitted version.

## Funding

This work was supported by grants from the Nature Science Foundation of China (No. 82002509, 81871967, 81903147), China Postdoctoral Science Foundation (No. 2019M661803, 2018M642223), Social Development Foundation of Jiangsu Province of China (No. BE2018604), Jiangsu Provincial Medical Talent, the Nanjing Science and Technology Project (No. 201803028).

## Conflict of Interest

The authors declare that the research was conducted in the absence of any commercial or financial relationships that could be construed as a potential conflict of interest.

## Publisher’s Note

All claims expressed in this article are solely those of the authors and do not necessarily represent those of their affiliated organizations, or those of the publisher, the editors and the reviewers. Any product that may be evaluated in this article, or claim that may be made by its manufacturer, is not guaranteed or endorsed by the publisher.

## Abbreviations

KB, ketone bodies; KD, ketogenic diet; KDT, Ketogenic dietary therapy; OXPHOS, oxidative phosphorylation; ssGSEA, Single-Sample Gene Set Enrichment Analysis; HCC, hepatocellular carcinoma; scRNA-seq, single cell RNA sequencing; TCGA, The Cancer Genome Atlas; GEO, Gene Expression Omnibus; CCLE, Cancer Cell Line Encyclopedia; ACC, adrenocortical carcinoma; BLCA, bladder urothelial carcinoma; LGG, brain lower grade glioma; BRCA, breast invasive carcinoma; CESC, cervical squamous cell carcinoma and endocervical adenocarcinoma; CHOL, cholangiocarcinoma; COAD, colon adenocarcinoma; ESCA, esophageal carcinoma; GBM, glioblastoma multiforme; HNSC, head and neck squamous cell carcinoma; KICH, kidney chromophobe; KIRC, kidney renal clear cell carcinoma; KIRP, kidney renal papillary cell carcinoma; LAML, acute myeloid leukemia; LIHC, liver hepatocellular carcinoma; LUAD, lung adenocarcinoma; LUSC, lung squamous cell carcinoma; DLBC, lymphoid neoplasm diffuse large B cell lymphoma; MESO, mesothelioma; OV, ovarian serous cystadenocarcinoma; PAAD, pancreatic adenocarcinoma; PCPG, pheochromocytoma and paraganglioma; PRAD, prostate adenocarcinoma; READ, rectum adenocarcinoma; SARC, sarcoma; SKCM, skin cutaneous melanoma; STAD, stomach adenocarcinoma; TGCT, testicular germ cell tumors; THYM, thymoma; THCA, thyroid carcinoma; UCS, uterine carcinosarcoma; UCEC, uterine corpus endometrial carcinoma; UVM, uveal melanoma.
