# Supplementary material for: Pan-Cancer Analysis of Glycolytic and Ketone Bodies Metabolic Genes: Implications for Response to Ketogenic Dietary Therapy
Source: Front Oncol. 2021 Oct 7;11:689068. doi: 10.3389/fonc.2021.689068 (PMC8529115; doi:10.3389/fonc.2021.689068)
Supplement: Supplementary file 1 [file DataSheet_1.docx]

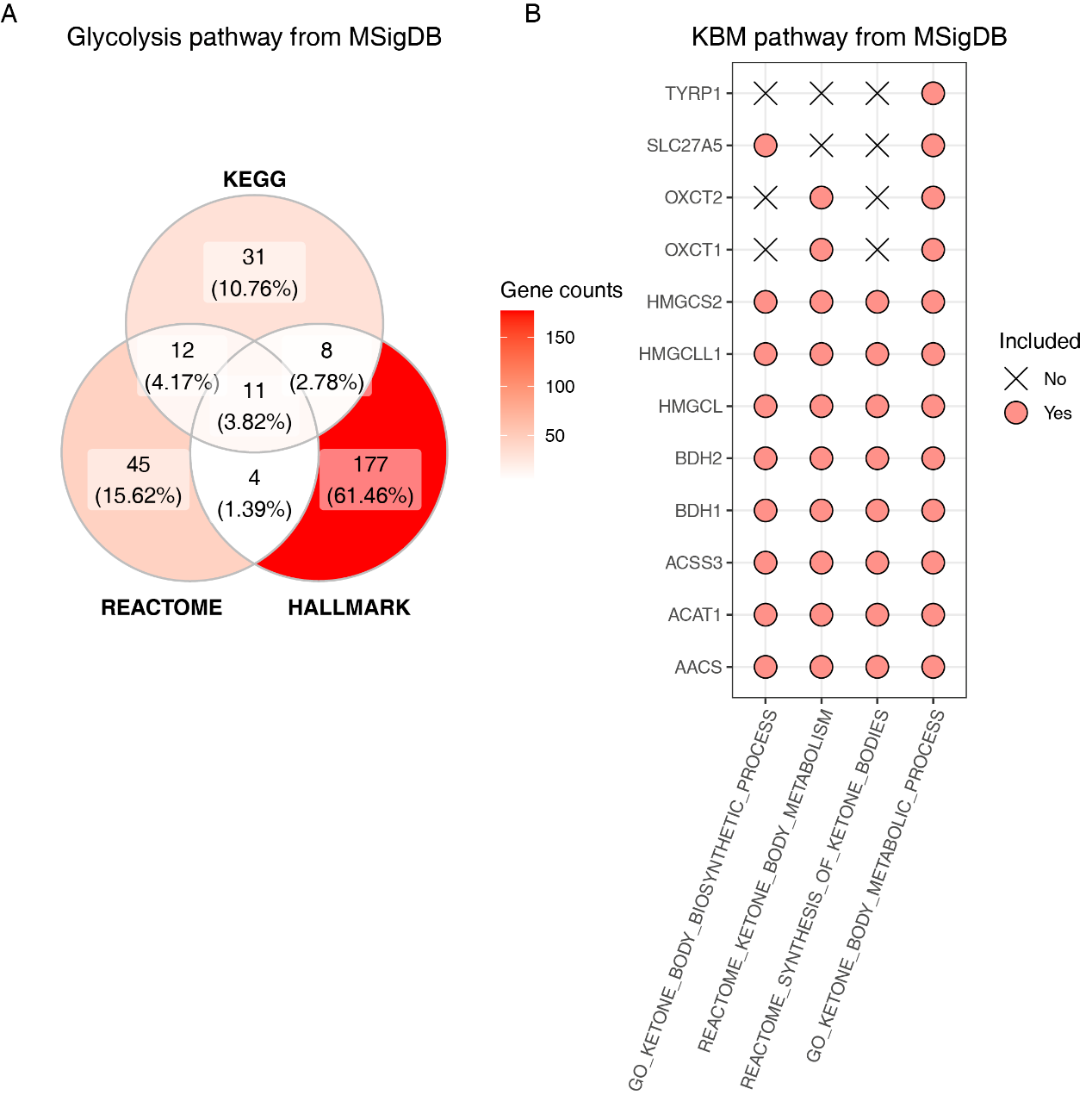


**Figure S1**. Determine the glycolytic and ketone body metabolic genes using MSigDB data. **(A)** The bubble plot shows the detailed genes in the curated ketone body metabolic pathway from the molecular signatures database (MSigDB). **(B)** Venn diagram showing the gene overlap among the glycolysis pathways from KEGG, Reactome and Hallmark gene sets.


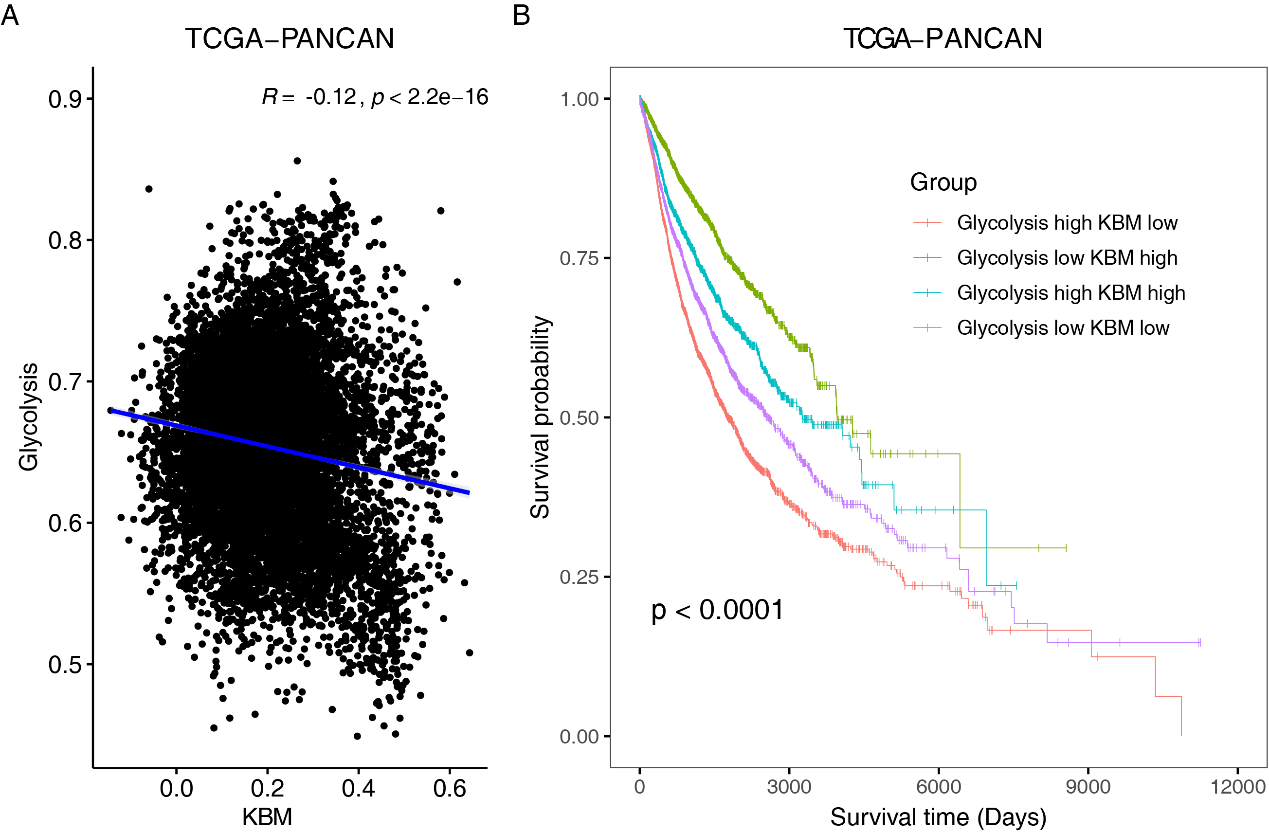


**Figure S2**. **(A)** The correlation between glycolysis and KBM in TCGA-PANCAN dataset based on ssGSEA score, which was measured by Spearman’s rank-order correlation. **(B)** The survival curve of differerent metabolic subtypes in TCGA-PANCAN dataset. The p value was determined by log-rank test.


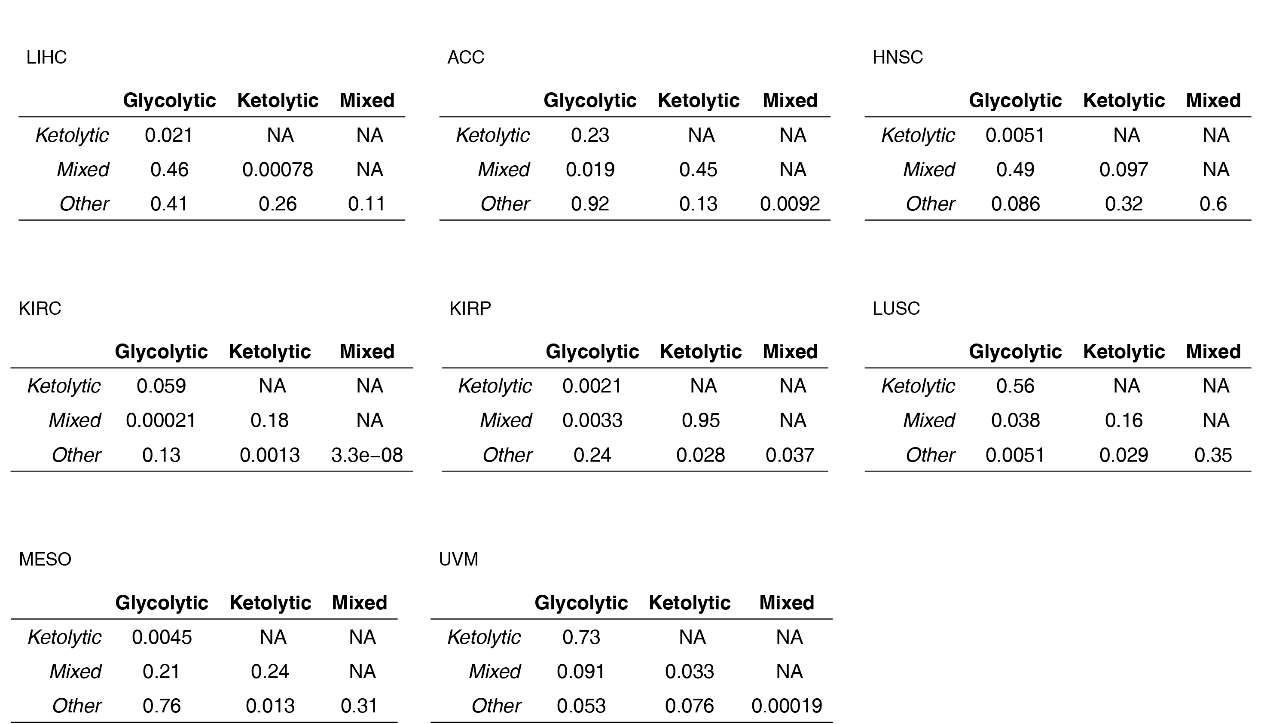


Figure S3. Kaplan-Meier analysis of overall survival of different cancer types stratified based on the metabolic subtypes. Logrank pvalues were shown in each pairwise comparison.


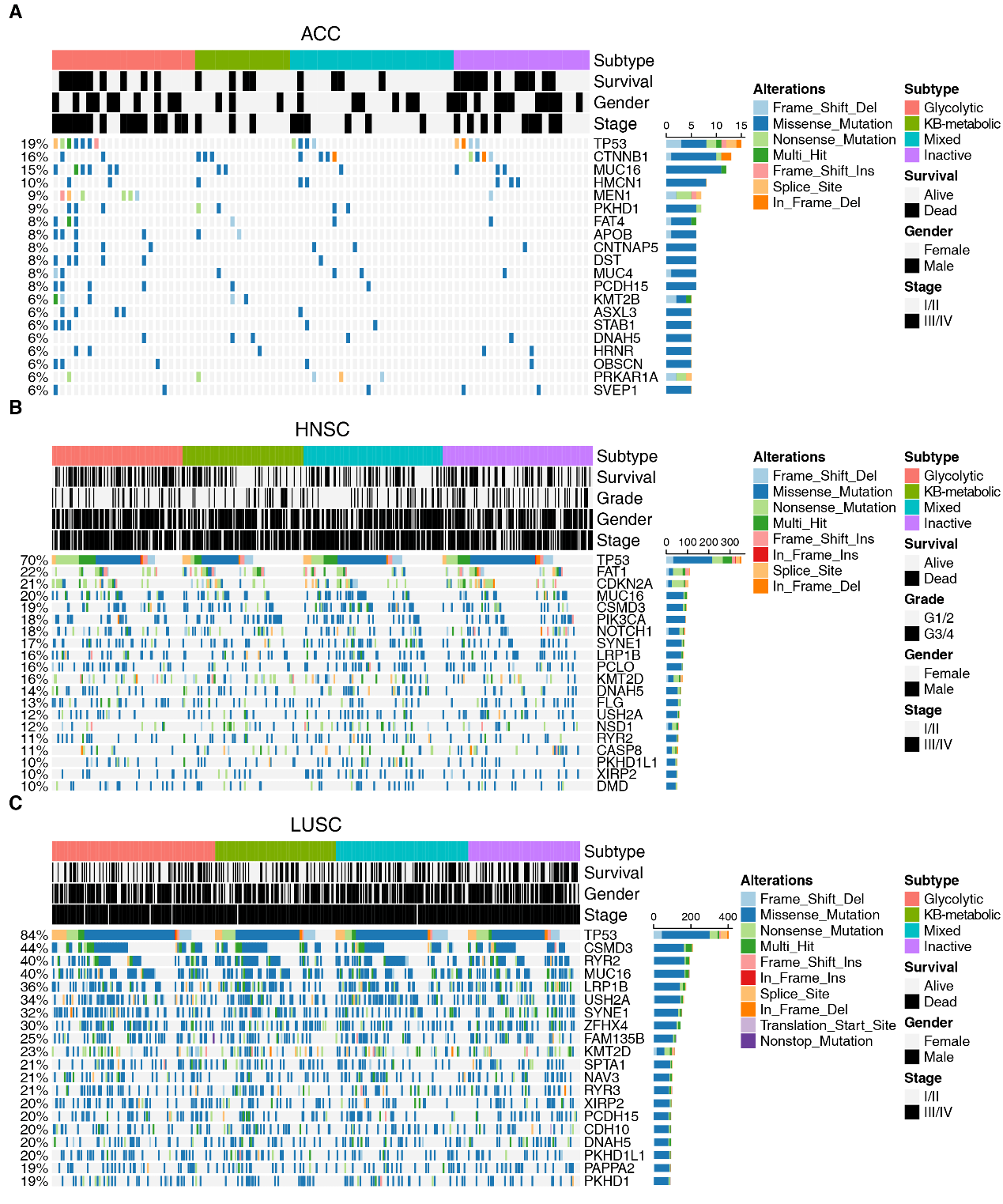


**Figure S4**. The mutational landscape of metabolic subtypes. Oncoplot showing the distribution of SNVs and INDELs mutational frequency in **(A)** ACC, **(B)** HNSC and **(C)** LUSC across the metabolic subtypes.
